# Effect of Nutritional Stress on the Expression of Sirtuin Gene Family

**DOI:** 10.1093/geroni/igaa057.407

**Published:** 2020-12-16

**Authors:** Xiaomin Zhang, Fathima Ameer, Jasmine Crane, Gohar Azhar, Jeanne Wei

**Affiliations:** 1 University of Arkansas for Medical Sciences, Little Rock, Arkansas, United States; 2 University of Arkansas for Medical Sciences, University of Arkansas for Medical Sciences, Arkansas, United States; 3 UAMS, Little Rock, Arkansas, United States

## Abstract

The sirtuin (SIRT) proteins are a highly conserved family of NAD+-dependent deacetylases that regulate histones as well as non-histone proteins. Seven sirtuin genes have been identified (SIRT1 to SIRT7) in mammals. SIRT1, SIRT6, and SIRT7 are primarily localized in the nucleus. SIRT2 is localized mainly in the cytoplasm. SIRT3, SIRT4, and SIRT5 are often located in the mitochondria. Therefore, the sirtuin family proteins exert their diverse functions at various cellular locations and regulate metabolism, stress responses, growth and aging processes. The sirtuin proteins are often considered as nutrient sensors. This study assessed the expression of sirtuin genes in C2C12 muscle cells under glucose stress conditions at different time points. Expression of all seven sirtuins was confirmed by Real-Time PCR analysis. SIRT1 (24 h) and SIRT3 (6 h and 15 h) are highly expressed under low glucose (2.7 mM) and high glucose (25 mM) conditions, whereas SIRT2 and SIRT4, SIRT5, SIRT6 and SIRT7 expressions were either relatively lower or there was no significant change under glucose stress conditions. Our results indicate that SIRT1 and SIRT3 demonstrated the greatest fluctuation in response to glucose stress, whether high or low glucose. These findings will help elucidate the role of sirtuins in the regulation of cellular processes, including metabolism. It will also help to enhance our understanding of the roles of sirtuin genes in regulation of blood sugar fluctuation in normal persons and diabetic patients, as well as in elderly individuals, many of whom are insulin resistant and “prediabetic” or diabetic.

